# Heparanase-2 protein and peptides have a protective effect on experimental glomerulonephritis and diabetic nephropathy

**DOI:** 10.3389/fphar.2023.1098184

**Published:** 2023-04-27

**Authors:** Baranca Buijsers, Marjolein Garsen, Mark de Graaf, Marinka Bakker-van Bebber, Chunming Guo, Xue Li, Johan van der Vlag

**Affiliations:** ^1^ Department of Nephrology, Radboud Institute of Molecular Life Sciences, Radboud University Medical Center, Nijmegen, Netherlands; ^2^ Departments of Urology and Pathology, Boston Children’s Hospital, Boston, MA, United States; ^3^ Department of Surgery, Harvard Medical School, Boston, MA, United States

**Keywords:** heparanase-1, heparanase-2, endothelial glycocalyx degradation, proteinuria, glomerulonephritis, diabetic nephropathy

## Abstract

**Introduction:** The endothelial glycocalyx degrading enzyme heparanase-1 (HPSE1) is a major contributor to kidney diseases, such as glomerulonephritis and diabetic nephropathy. Therefore, inhibition of HPSE1 could be an interesting therapeutic strategy to treat glomerular diseases. A possible HPSE1 inhibitor is heparanase-2 (HPSE2) because HPSE2 is a structural homolog of HPSE1 without enzymatic activity. The importance of HPSE2 has been recently demonstrated in HPSE2-deficient mice, since these mice developed albuminuria and died within a few months after birth. We postulate that inhibition of HPSE1 activity by HPSE2 is a promising therapeutic strategy to target albuminuria and resulting renal failure.

**Methods:** First, we evaluated the regulation of HPSE2 expression in anti-GBM and LPS-induced glomerulonephritis, streptozotocin-induced diabetic nephropathy, and adriamycin nephropathy by qPCR and ELISA. Second, we measured the HPSE1 inhibiting capacity of HPSE2 protein and 30 different HPSE2 peptides and assessed their therapeutic potential in both experimental glomerulonephritis and diabetic nephropathy using kidney function and cortical mRNA expression of HPSE1 and cytokines as outcome parameters.

**Results:** HPSE2 expression was downregulated under inflammatory and diabetic conditions, whereas this effect on HPSE2 expression was absent with HPSE1 inhibition and in HPSE1-deficient mice. Both HPSE2 protein and a mixture of the three most potent HPSE1 inhibitory HPSE2 peptides could prevent LPS and streptozotocin induced kidney injury.

**Discussion:** Taken together, our data suggest a protective effect of HPSE2 in (experimental) glomerular diseases and support the therapeutic potential of HPSE2 as HPSE1 inhibitor in glomerular diseases.

## Introduction

Diabetic nephropathy (DN) and glomerulonephritis are two distinct types of glomerular diseases that affect millions of people worldwide. A common hallmark of DN and glomerulonephritis is proteinuria. Various studies have shown that a key player in the development of proteinuria in glomerular diseases is the endothelial glycocalyx (Garsen et al., 2014; van der Vlag and Buijsers, 2020). The endothelial glycocalyx is a network of glycoproteins including glycosaminoglycans (GAGs) attached to membrane-bound proteoglycans covering the endothelial cells, which is a crucial component of the glomerular filtration barrier (GFB) (Singh et al., 2007; Fridén et al., 2011). Moreover, the main sulphated GAG in the endothelial glycocalyx is heparan sulfate (HS). HS has diverse functions, including charge-selective permeability of the GFB within the glycocalyx, binding of several ligands, such as growth factors, cytokines, and chemokines, and mediating trafficking of leukocytes under inflammatory conditions (Goldberg et al., 2013). Many glomerular diseases are characterized by reduced levels of HS in the GFB, and this reduced HS expression has been associated with increased glomerular expression of heparanase-1 (HPSE1) and albuminuria (van der Vlag and Buijsers, 2020). HPSE1 is the only mammalian enzyme that hydrolyses heparan sulfate (HS). Notably, we have previously shown that HPSE1 is essential in the development of albuminuria and renal damage in both experimental DN and experimental glomerulonephritis (Gil et al., 2012; Garsen et al., 2016a).

Current treatment of patients with DN and glomerulonephritis includes blockade of the renin-angiotensin-aldosterone system (RAAS) with ACE inhibitors or treatment with immunosuppressive drugs, respectively (Jefferson, 2018; Yamazaki et al., 2021). However, these drugs do not sufficiently reduce albuminuria and renal damage or induce severe adverse effects, thus justifying the discovery of additional treatment options. Inhibition of HPSE1 activity represents an excellent therapeutic target to reduce albuminuria and renal damage. Drugs that inhibit HPSE1 can be roughly divided in two categories, HS-mimetics (Hammond et al., 2013), which are drugs that structurally resemble HS, and inhibitors that directly block the HS-binding site on HPSE1. Currently, a few heparin-based HPSE1 inhibitors are in development. A previous study showed that the heparin-based HS mimetic SST0001 decreased albuminuria 2-fold in experimental type 1 and 2 DN (Gil et al., 2012).

A major draw-back of heparin-based HPSE1 inhibitors is the simultaneous activation of macrophages via Toll-like receptors (Goldberg et al., 2014). To overcome this problem, there is an unmet need for novel HPSE1-inhibiting compounds that are not able to activate macrophages. The structural homolog of HPSE1, heparanase- 2 (HPSE2), might embody such a novel compound. The importance of HPSE2 was stressed by a recent study in which it was demonstrated that HPSE2-deficient mice develop a significant albuminuria and die within 1 month after birth (Guo et al., 2015). This study suggests that HPSE2 serves as a natural break to control HPSE1 activity. HPSE2 shares 44% identity and 59% similarity with HPSE1 even though HPSE2 has no enzymatic activity like HPSE1 (Levy-Adam et al., 2010). Both HPSE1 and HPSE2 bind to cell associated HS proteoglycans and, therefore, are competitors. However, unlike HPSE1, HPSE2 fails to get internalized and instead binds with high affinity to HS and remains on the cell surface. In addition, HPSE2 can physically interact with HPSE1, thereby preventing cleavage of HS chains (Levy-Adam et al., 2010). Both mechanisms may contribute to the potential of HPSE2 to inhibit HPSE1 activity. Finally, an important feature of HPSE2 compared to GAG-based compounds is that HPSE2 inhibits HPSE1 without activating macrophages unlike GAG-based compounds.

Although Kiyan et al. (2019) proved the protective effect of HPSE2 protein for LPS induced HPSE1-mediated microvascular inflammation during the course of our study, the potential of HPSE2 as an inhibitor of HPSE1 activity in glomerular diseases has not been investigated yet.

In this study, we aimed to investigate whether inhibition of HPSE1 activity by HPSE2 is a promising therapeutic strategy to target albuminuria and renal failure associated with glomerular diseases. Therefore, we evaluated the regulation of HPSE2 expression under inflammatory and diabetic conditions and tested the therapeutic potential of HPSE2 protein and HPSE2-derived peptides in both experimental glomerulonephritis and experimental DN.

## Materials and methods

### Animals

Eight-to twelve-week-old C57Bl/6J wild-type (wt) mice (Charles River, Germany) were kept under pathogen-free conditions and housed in a temperature-controlled room with a 12-h light/dark cycle with *ad libitum* access to food and water. All experiments were approved by the Animal Ethical Committee of the Radboud University Nijmegen.

Tissue, urine, and plasma were obtained from HPSE2 knockout C57Bl/6J mice, wt and HPSE1-deficient C57Bl/6J mice eventually with induced experimental glomerulonephritis and experimental type 1 DN, adriamycin nephropathy wt C57Bl/6J mice, and SST0001 treated type 2 DN in obese db/db mice, as described previously (Gil et al., 2012; Guo et al., 2015; Garsen et al., 2016a; Garsen et al., 2016b).

### Experimental disease models

Experimental LPS-induced glomerulonephritis was induced in eight-to twelve-week-old wt C57Bl/6J mice by an i. p. injection with 80 µg LPS (O111:B4; Sigma-Aldrich Chemie, Zwijndrecht, Netherlands) as described previously (Garsen et al., 2016a). The mice were treated with heparanase 2 (HPSE2) protein and an equal mix of HPSE2 peptides (Thermo Fisher Scientific, Breda, Netherlands) 381–410, 391–420, and 401–430, amino acids respectively, via repeated i. v injection with 12 h intervals starting 6 h after LPS injection. Mice were sacrificed 48 h after LPS injection.

Experimental streptozotocin (STZ)-induced type 1 diabetes was induced in six-to eight-week-old wt DBA/2J mice (Charles River, Germany). Mice were first fasted for 4 h whereafter the mice were injected i. p. with 50 mg/kg STZ (Sigma-Aldrich Chemie, Zwijndrecht, Netherlands) in 250 mM sodium citrate buffer (pH 4.5), whereas controls were injected with sodium citrate buffer alone for 5 consecutive days. Blood glucose levels were measured twice a week using the ACCU-CHEK Aviva Blood Glucose Meter (Roche Diagnostics, Almere, Netherlands) to monitor that blood glucose levels would not raise above 25 mmol/L. Mice were implanted under the skin half an insulin pellet (LinShin Canada, Toronto, Canada) when their blood glucose level did raise above 25 mmol/L. The mice were treated with HPSE2 protein via continuous s. c infusion with an osmotic mini pump 8 weeks after the final injection with STZ for a duration of 4 weeks. Mice were sacrificed 12 weeks after induction of diabetes.

Kidneys were collected and snap frozen in liquid nitrogen and urine was collected after 18 h housing in metabolic cages upon termination. Urinary albumin was measured by radial immunodiffusion (Mancini) as described previously (Garsen et al., 2016a). Blood urea nitrogen (BUN) and urinary/plasma creatinine concentrations were determined routinely in our clinical diagnostic facility.

### RNA isolation and real-time PCR

RNA was isolated from the kidney cortex using RNeasy mini kit (Qiagen) and 1 µg of RNA was reverse transcribed into cDNA using the Transcription First Strand cDNA synthesis kit (Roche, Woerden, Netherlands) according to manufacturer’s instructions. Quantitative PCR was performed with SYBR Green (Roche, Woerden, Netherlands) on a CFX 96 C1000 Thermal Cycler (Bio-rad, Lunteren, Netherlands) to determine expression levels. Data was analysed using glyceraldehyde-3-phosphate dehydrogenase (GAPDH) as housekeeping gene and by making use of the delta-delta CT method. Sequences of gene-specific primers are listed in Table 1.

**TABLE 1:** Primers used in real-time PCR.

Target	Primer sequence
GAPDH	F: 5′-AGA​AAC​CTG​CCA​AGT​ATG​ATG​AC-3′
	R: 5′-TCA​TTG​TCA​TAC​CAG​GAA​ATG​AG-3′
HPSE2	F: 5′-GCA​CCA​AGA​ACC​CAG​TCA​GG-3′
	R: 5′-ATC​ACT​CCG​GAC​AAT​GTC​ATC​C-3′

F, forward; R, reverse; HPSE2, heparanase-2; GAPDH, glyceraldehyde-3-phosphate dehydrogenase.

### *In vitro* translation of HPSE2 protein

HPSE2 protein had to be produced to test the hypothesis that HPSE2 can be used to attenuate the induction of glomerulonephritis and DN. Unfortunately, we were not able to make stable cell lines and therefore initiated the production of HPSE2 with an *in vitro* translation (IVT) system.

The pT7CFE1 N-His vector was used for cloning of the mHPSE2 gene. Standard protocols were followed for DNA manipulation. Enzymes used for endonuclease digestions, backbone dephosphorylation and ligations were retrieved from New England Biolabs (NEB, Hitchin, United Kingdom). mHPSE2 primer sets were designed based on the cDNA sequence of the mHPSE2 gene (Genbank accession number: NM_001081257). Template material, FLAG-mHPSE2 pcDNA3.1, was kindly provided by professor Dr. Israel Vlodavsky (Technion-Israel Institute of Technology, Haifa, Israel). PCR was used for the amplification of the mHPSE2 gene using a MyIQ^®^ PCR System (Bio-Rad, Veenendaal, Netherlands) and Platinum^®^ PCR SuperMix (Invitrogen, Landsmeer, Netherlands) with the primers listed in Table 2 according to the manufacturer’s recommendations. mHPSE2 PCR fragments were purified from agarose gels with a gel extraction kit (Qiagen, Hilden, Germany) for cloning purposes.

**TABLE 2:** Primers used in cloning experiment.

Target	Primer sequence
mHPSE2	F: 5′-AGG​CGA​ATT​CGC​CAC​CAT​GAG​GGT​GCT​CTG​TGC​TTT​C-3′
	R: 5′-AAT​CTC​GAG​TCG​ATA​ACG​GCA​GGC​C-3′

F, forward; R, reverse; HPSE2, heparanase-2.

The mHPSE2 gene was cloned into the EcoRI and XhoI sites of pT7CFE1 N-His using restriction endonuclease digestion and ligation of the respective fragments. IVT was performed in a dialysis device according to manufacturer’s instructions (Thermo Fisher Scientific, cat# 88891). The IVT reactions were prepared according to the manufacturer’s instructions as well. Briefly, HeLa lysate was incubated with accessory proteins followed by the addition of the provided reaction mix, nuclease-free water, RNAse inhibitor and 4 μg plasmid DNA (His-mHPSE2 pT7CFE1). The plasmid pCFE-GFP was used as a positive control for protein translation. Next, the mixture was centrifuged at 10,000 g, and the supernatant was transferred to the dialysis device and placed in an incubator which was set to 30°C for 6 h. After incubation, the resulting reactions were collected from the dialysis device and directly used for His tag-mediated affinity purification or for long-term storage at −20°C. Schematic representation of the final construct His-mHPSE2 pcDNA3.1 is depicted in Supplementary Figure 1A.

### Western blot analysis

Purified HPSE2 was mixed with sample buffer containing SDS and β-mercaptoethanol, whereupon it was heated at 95°C and resolved by electrophoresis under reducing conditions on a 10% SDS-polyacrylamide gel. The resolved proteins were transferred to an Amersham Protran 0.45 μm NC nitrocellulose membrane (GE Healthcare Life science, Freiburg, Germany) by blotting at 100 V for 1.5 h at 4°C. Hereafter, the membranes were blocked with blocking solution (Roche, Woerden, Netherlands) for 1 h at room temperature (RT). The membranes were incubated for 1 h in PBGTNa solution (PBS containing 0.5% bovine serum albumin, 0.05% gelatin, 0.05% Tween-20 and 300 mM NaCl) with primary antibodies (Mouse anti-HIS (Sigma-Aldrich Cat# H1029, RRID:AB_260015) 1:4,000 and rabbit anti-HPA2 (Thermo Fisher Scientific Cat# PA5-21326, RRID:AB_11153273) 1:2,000) followed by washing with PBS containing 0.05% tween-20. Subsequently, the membranes were incubated for 1 h with the appropriate peroxidase conjugated secondary antibody 1:10,000 diluted in PBGTNa solution (peroxidase AffiniPure goat anti-mouse IgG (H + L) and peroxidase AffiniPure donkey anti-rabbit IgG (H + L) respectively (Jackson ImmunoResearch Laboratories, West Grove, PA). The membranes were washed with PBS containing 0.05% tween-20. Resolved proteins were detected by chemiluminescence using Pierce™ ECL pico Western blotting reagent (Thermo Fisher Scientific, Breda, Netherlands). Western blot images were obtained using the ChemiDoc™ XRS + imaging system (Bio-Rad, Veenendaal, Netherlands) (Supplementary Figures 1B, C).

### HPSE1 activity assay

The activity of HPSE1 in renal cortex was measured using a commercially available HPSE1 assay kit (AMS Biotechnology, Abingdon, United Kingdom, Cat#Ra001-BE-K) according to the manufacturer’s instruction.

The inhibition of HPSE1 activity by HPSE2 protein and peptides was determined by the commercially available HPSE1 assay kit and an in-house developed HPSE1 activity assay, which was optimized by the use of recombinant active human HPSE1 (Bio-techne, Abingdon, United Kingdom, Cat#7570-GH-005). The in-house developed activity assay was performed as described previously (Buijsers et al., 2020). In short, Nunc maxisorp flat bottom 96 plates (Thermo scientific, Breda, Netherlands) were coated with 10 μg/mL heparan sulfate from bovine kidney (HSBK) (Sigma-Aldrich, Zwijndrecht, Netherlands) in optimized HS coating buffer, overnight in a humidified chamber at RT. Subsequently, plates were blocked for minimal 2 h with 1% bacto-gelatin (Difco laboratories, Detroit, Michigan, United States) in PBS at RT whereupon HPSE1 inhibitors were added to the plate with a standard amount of recombinant HPSE1 in HPSE1 buffer. Remaining HSBK was detected with primary mouse anti-rat IgM HS antibody JM403 (Amsbio, Abingdon, United Kingdom, cat. No. #370730-S, RRID: AB_10890960, 1 μg/mL in PBST) for 1 h at RT. Subsequently, plates were incubated with secondary goat anti-mouse IgM HRP antibody (Southern Biotech, Uden, Netherlands, cat. No. #1020–05, RRID: AB_2794201, dilution 1:10,000 in PBST) for 1 h at RT. Finally, 3,3′,5,5′-tetramethylbenzidine (TMB) substrate (Invitrogen, Breda, Netherlands) was added and reaction was stopped by addition of 2 M sulfuric acid, and absorbance was measured at 450 nm.

### HPSE2 ELISA

HPSE2 expression was determined with a commercially available HPSE2 detection ELISA (Bio-Connect, Huissen, Netherlands Cat#CSB-EL010717HU) according to manufacturer’s instruction.

### Immunofluorescence staining

Glomerular heparan sulfate expression was determined by immunofluorescence staining on 2 µm sections of fresh-frozen kidneys. The primary antibody that was used to visualize heparan sulfate was scFv HS4C3 (VSV) (in house produced) and as secondary antibody anti VSV-G-CY3 (Sigma, cat#C7706) was used. Staining intensity of heparan sulfate was scored in 30 glomeruli per section on a scale between 0 (no staining) and 10 (maximal staining). Scoring was performed on blinded sections by two independent investigators.

### Statistical analyses

Values are expressed as mean ± SEM. Shapiro-Wilk test or Kolmogorov-Smirnov test was performed to test for normality of data. Significance was determined by Student’s t-test or Mann Whitney test to compare two groups and by one-way ANOVA followed by Dunnett’s test or Kruskal–Wallis test followed by Dunn’s test to compare more than two groups. All analyses were performed using GraphPad Prism V.9.1.2 (La Jolla, United States). *p* values less than 0.05 were considered as statistically significant.

## Results

### Induction of experimental glomerular diseases causes downregulation of endogenous HPSE2 expression

**FIGURE 1:**
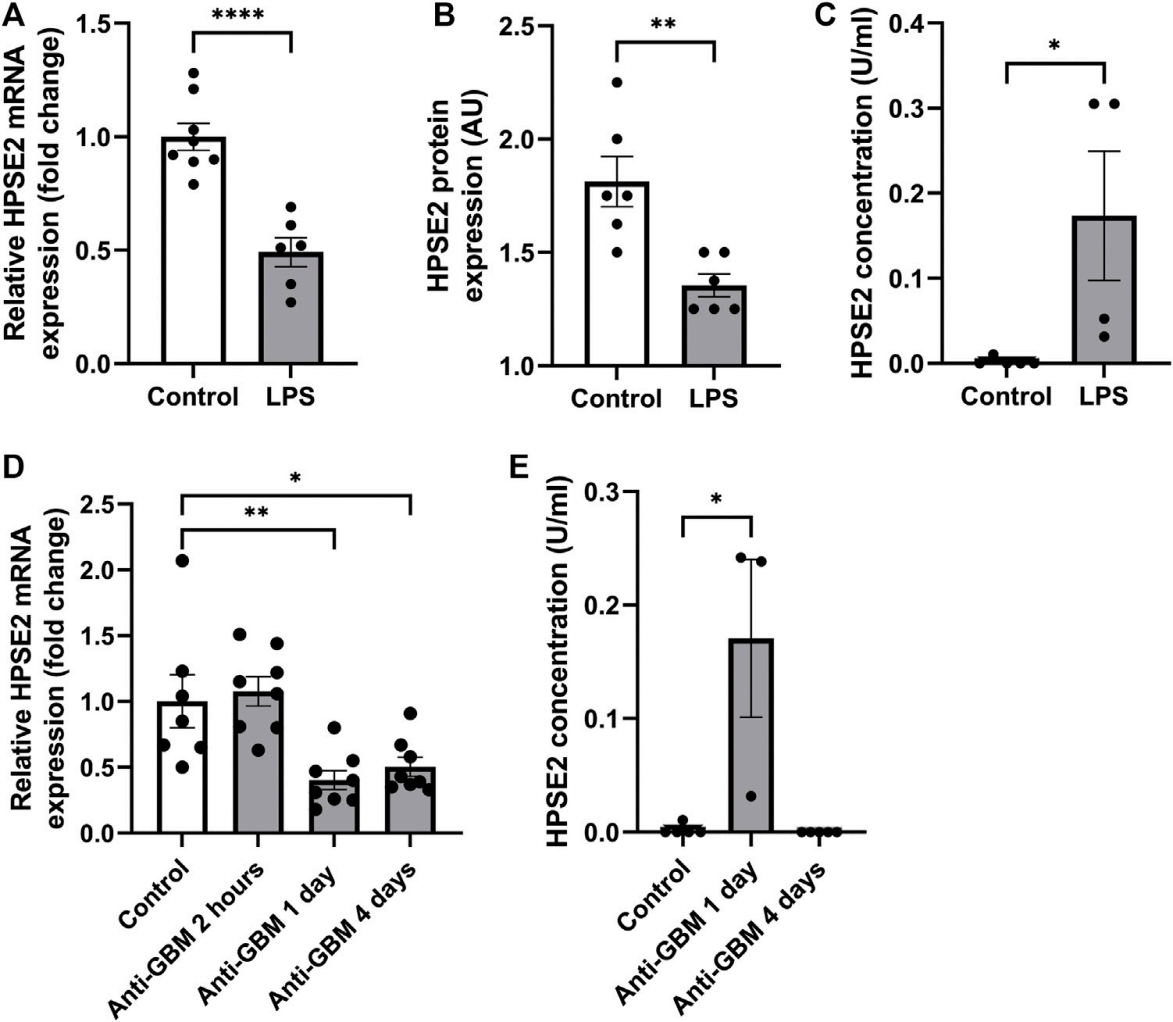
HPSE2 is downregulated in experimental glomerulonephritis **(A)**. Renal cortical HPSE2 mRNA expression of wild-type mice treated with or without LPS for 2 days **(B)**. Renal cortical HPSE2 protein expression of wild-type mice treated with or without LPS for 2 days **(C)**. HPSE2 concentration in urine of wild-type mice treated with or without LPS for 2 days **(D)**. Renal cortical HPSE2 mRNA expression of wild-type mice treated with or without anti-GBM for 2 h, 1 day or 4 days **(E)**. HPSE2 concentration in urine of wild-type mice treated with or without anti-GBM for 1 day or 4 days. Data are expressed as mean ± SEM. *n* ≥ 3 *p < 0.05, **p < 0.01, ****p < 0.0001. HPSE2, heparanase-2; AU, arbitrary units; GBM, glomerular basement membrane.

**FIGURE 2:**
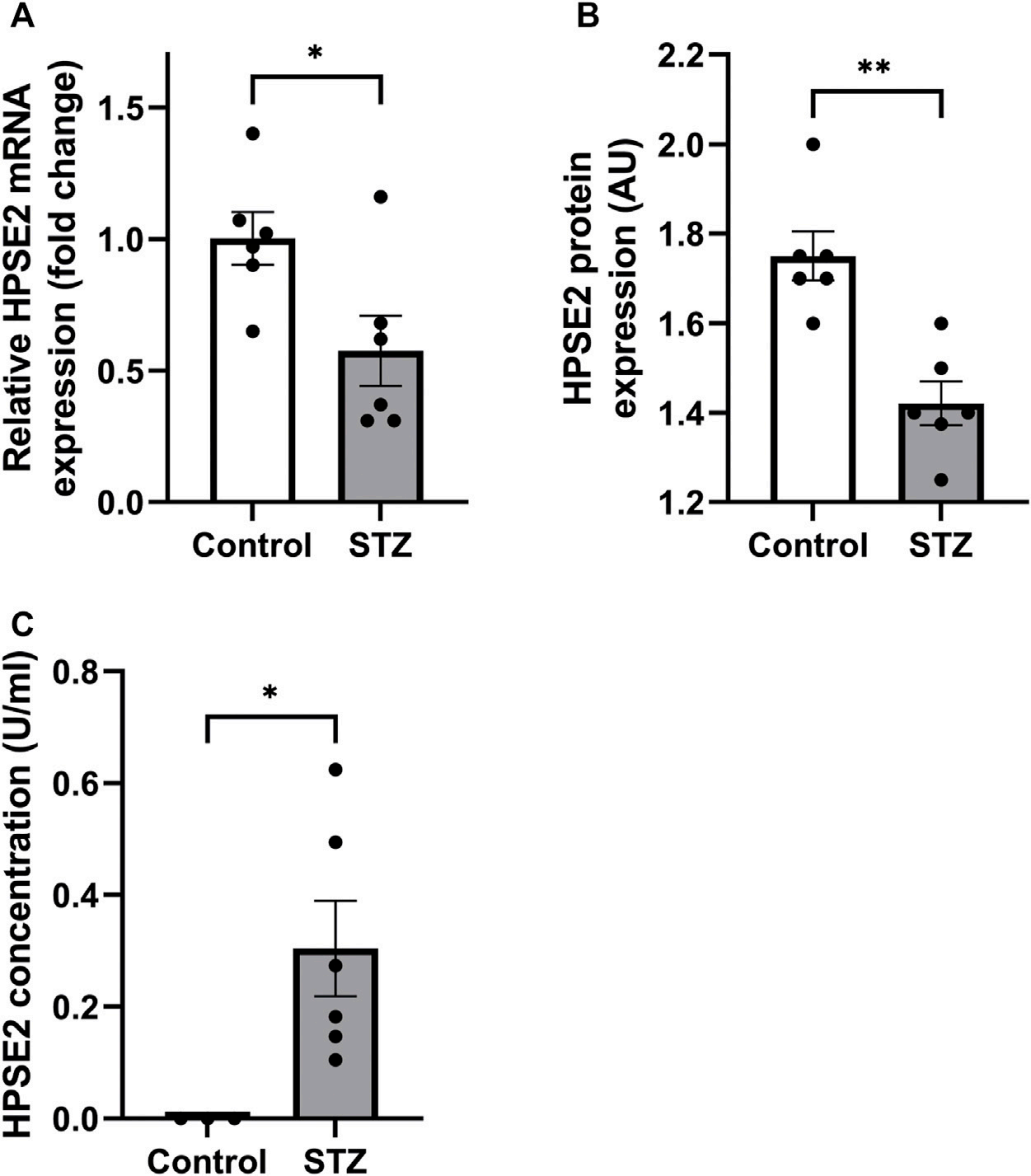
HPSE2 is downregulated in experimental diabetic nephropathy **(A)**. Renal cortical HPSE2 mRNA expression of wild-type DBA/2J mice treated with or without STZ for 16 weeks **(B)**. Renal cortical HPSE2 protein expression of wild-type mice treated with or without STZ for 16 weeks **(C)**. HPSE2 concentration in urine of wild-type mice treated with or without STZ for 16 weeks. Data are expressed as mean ± SEM. *n* ≥ 3 *p < 0.05, **p < 0.01. HPSE2, heparanase-2; AU, arbitrary units; STZ, streptozotocin.

Recently, Kiyan et al. (2019) showed decreased HPSE2 expression in serum of mice induced with cecal ligation and puncture (CLP) polymicrobial sepsis model compared to control mice. Here, we quantified HPSE2 expression in the renal cortex during experimental glomerular diseases to gain a better understanding of the regulation of endogenous HPSE2. In line with the finding from Kiyan et al., decreased mRNA and protein expression of HPSE2 were detected in the renal cortex upon LPS treatment (Figures 1A, B). Moreover, LPS-induced glomerulonephritis in mice was associated with higher HPSE2 secretion in urine (Figure 1C), which might be ascribed to the increased filtration. Similarly, HPSE2 mRNA expression was decreased in anti-GBM induced glomerulonephritis after one and 4 days, but not yet after 2 hours (Figure 1D), and urinary secretion of HPSE2 (Figure 1E) was increased 1 day after anti-GBM administration. We observed similar effects on HPSE2 regulation in streptozotocin-induced DN. Both HPSE2 mRNA and protein expression were downregulated in STZ-induced DN mice (Figures 2A, B), while urinary HPSE2 secretion was upregulated (Figure 2C). The effect on HPSE2 regulation was not limited to glomerulonephritis and DN since downregulation of HPSE2 mRNA and protein expression could be observed in adriamycin nephropathy, a model for FSGS, as well (Supplementary Figures 1A, B). These results collectively indicate a decreased HPSE2 expression during different experimental glomerular diseases.

### HPSE1 suppression affects expression of endogenous HPSE2 in experimental glomerular diseases

**FIGURE 3:**
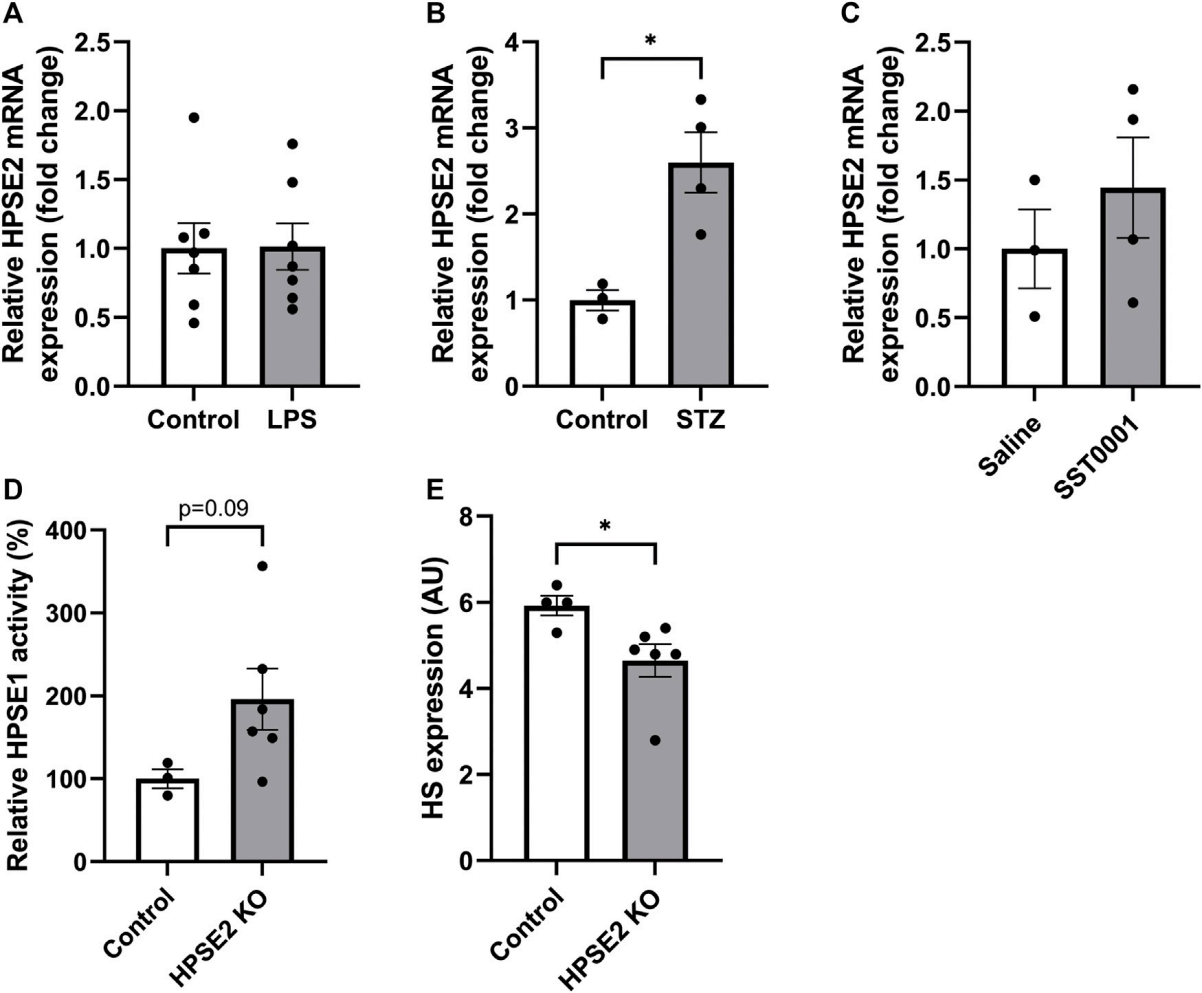
Relation between HPSE1 and HPSE2 regulation in glomerular disease reveals a possible connection **(A)**. Renal cortical HPSE2 mRNA expression of HPSE1 KO mice treated with or without LPS for 2 days **(B)**. Renal cortical HPSE2 protein expression of HPSE1 KO mice treated with or without STZ for 16 weeks **(C)**. Renal cortical HPSE2 mRNA expression of type 2 diabetic mice treated with HPSE1 inhibitor SST0001 or left untreated **(D)**. Relative HPSE1 activity of untreated wild-type mice or HPSE2 KO mice **(E)**. Glomerular HS expression of untreated wild-type mice or HPSE2 KO mice. Data are expressed as mean ± SEM. *n* ≥ 3 *p < 0.05. HPSE2, heparanase-2; AU, arbitrary units; HPSE1, heparanase-1; KO, knock-out; STZ, streptozotocin.

We investigated whether the observed decreased expression of HPSE2 can be attributed to increased levels of HPSE1, since HPSE1 expression and activity are increased in glomerular diseases. (Gil et al., 2012; Garsen et al., 2016a; van der Vlag and Buijsers, 2020). In contrast to wild-type mice, HPSE2 expression was not downregulated in LPS-induced glomerulonephritis in HPSE1-deficient mice (Figure 3A). In fact, HPSE2 expression was even upregulated in STZ-induced DN in HPSE1-deficient mice (Figure 3B). Similarly, DN type 2 mice treated with the HPSE1 inhibitor SST0001 showed a trend of increased HPSE2 expression (Figure 3C). Apparently, the effect on HPSE2 regulation in experimental glomerular diseases seems to be reversed by inhibition or lack of HPSE1 expression and activity.

The importance of HPSE2 has been demonstrated by HPSE2-deficient mice. In a recent study, HPSE2-deficient mice were reported to suffer from growth retardation and death within 1 month after birth. (Guo et al., 2015). Analysis of the renal cortex from these HPSE2-deficient mice showed increased HPSE1 activity (Figure 3D), which was accompanied by a decrease in glomerular HS expression (Figure 3E). Collectively, these findings indicate that reversing the observed decrease in endogenous HPSE2 expression in glomerular diseases can be an interesting therapeutic strategy, which may be mediated by HPSE1 inhibition.

### HPSE2 protein and peptides inhibit HPSE1 activity

**FIGURE 4:**
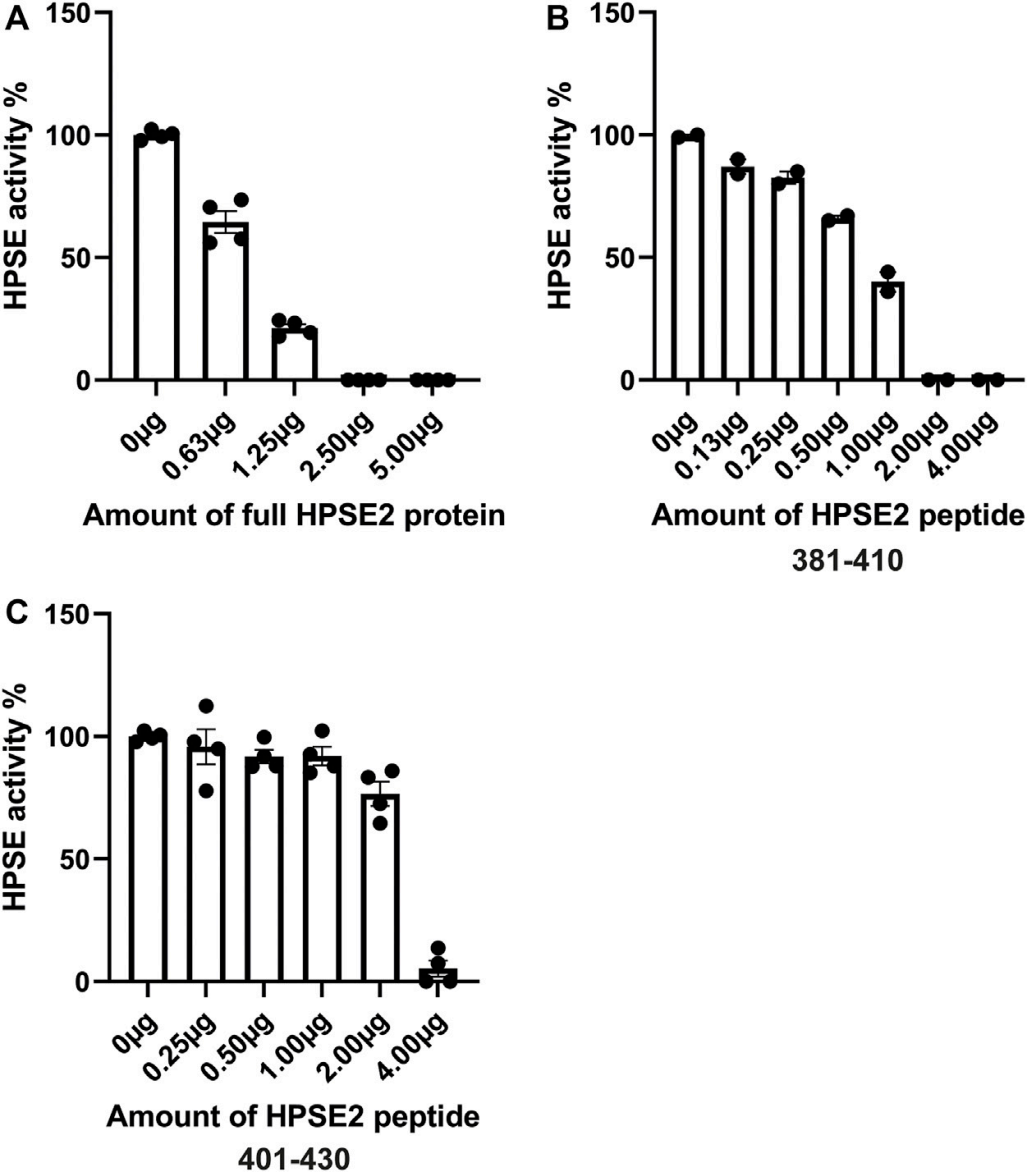
Inhibition of HPSE1 activity is dose-dependently achieved by HPSE2 protein and HPSE2 peptides. Dose response inhibition of recombinant human HPSE1 with the full HPSE2 protein **(A)** and HPSE2 peptides **(B,C)**. HPSE2, heparanase-2; HPSE1, heparanase-1.

Previous studies have shown the ability of HPSE2 to inhibit HPSE1. However, the mechanisms behind HPSE2 mediated HPSE1 inhibition are still incompletely understood. In this study, we aimed to further explore the possibility of HPSE2 as therapeutic to target HPSE1 mediated glomerulonephritis and DN. We produced HPSE2 protein by an *in vitro* translation system and designed 30 different overlapping HPSE2 peptides (Table 3). In a first screening experiment of all 30 HPSE2 peptides, HPSE2 peptide 381–410 and HPSE2 peptide 401–430 showed great promise as inhibitor of HPSE1 activity (data not shown). Notably, the in house produced full-length HPSE2 protein showed a dose-dependent inhibition of HPSE1 activity (Figure 4A). The HPSE2 peptides inhibited HPSE1 activity in a dose dependent manner as well (Figures 4B, C). Since HPSE2 peptide 381–410 and HPSE2 peptide 401–430 have overlapping sequences, we designed one additional peptide, HPSE2 peptide 391–420, to evaluate.

**TABLE 3:** Sequences of the designed HPSE2 peptides.

Protein	AA region	Peptide sequence
HPSE2	1–30	MRVLCAFPEAMASSSSRPPSCLALVALFLA
HPSE2	17–46	RPPSCLALVALFLALLLHLSLSFHAGNRRP
HPSE2	41–70	AGNRRPLPVDRATGLKEKTLILLDVSTKNP
HPSE2	61–90	ILLDVSTKNPVRTVNENFLSLQLDPSIIHD
HPSE2	81–110	LQLDPSIIHDGWLDFLSSKRLVTLARGLSP
HPSE2	101–130	LVTLARGLSPAFLRFGGKRTDFLQFQNLRN
HPSE2	121–150	DFLQFQNLRNPAKSRGGPGPDYYLKNYEDD
HPSE2	141–170	DYYLKNYEDDIVRSDVALDKQKGCKIAQHP
HPSE2	161–190*	QKGCKIAQHPDVMLELQREKASQMHLVLLK
HPSE2	181–210*	ASQMHLVLLKEQYSNTYSNLILTARSLDKL
HPSE2	201–230*	ILTARSLDKLYNFADCSGLHLIFALNALRR
HPSE2	221–250	LIFALNALRRNPNNSWNSSSALSLLKYSAS
HPSE2	241–270*	ALSLLKYSASKKYNISWELGNEPNNYRSIH
HPSE2	261–290	NEPNNYRSIHGRAVNGSQLGKDYIQLKSLL
HPSE2	281–310	KDYIQLKSLLQPIRVYSRASLYGPNIGRPR
HPSE2	301–330	LYGPNIGRPRKNVIALLDGFMKVAGSTVDA
HPSE2	321–350*	MKVAGSTVDAVTWQHCYIDGRVVKVMDFLK
HPSE2	341–370	RVVKVMDFLKTRLLDTLSDQIRKIQKVVNT
HPSE2	361–390	IRKIQKVVNTYTPGKKIWLEGVVTTSAGGT
HPSE2	381–410	GVVTTSAGGTNNLSDSYAAGFLWLNTLGML
HPSE2	401–430	FLWLNTLGMLANQGIDVVIRHSFFDHGYNH
HPSE2	421–450	HSFFDHGYNHLVDQNFNPLPDYWLSLLYKR
HPSE2	441–470*	DYWLSLLYKRLIGPKVLAVHVAGLQRKPRP
HPSE2	461–490	VAGLQRKPRPGRVIRDKLRIYAHCTNHHNH
HPSE2	481–510*	YAHCTNHHNHNYVRGSITLFIINLHRSRKK
HPSE2	501–530	IINLHRSRKKIKLAGTLRDKLVHQYLLQPY
HPSE2	521–550	LVHQYLLQPYGQEGLKSKSVQLNGQPLVMV
HPSE2	541–570	QLNGQPLVMVDDGTLPELKPRPLRAGRTLV
HPSE2	551–580	DDGTLPELKPRPLRAGRTLVIPPVTMGFYV
HPSE2	563–592*	LRAGRTLVIPPVTMGFYVVKNVNALACRYR

Peptides indicated with a * precipitated and could thus not be tested. HPSE2, heparanase-2; AA, amino-acid.

### HPSE2 protein and peptides ameliorate the induction of glomerulonephritis *in vivo*

**FIGURE 5:**
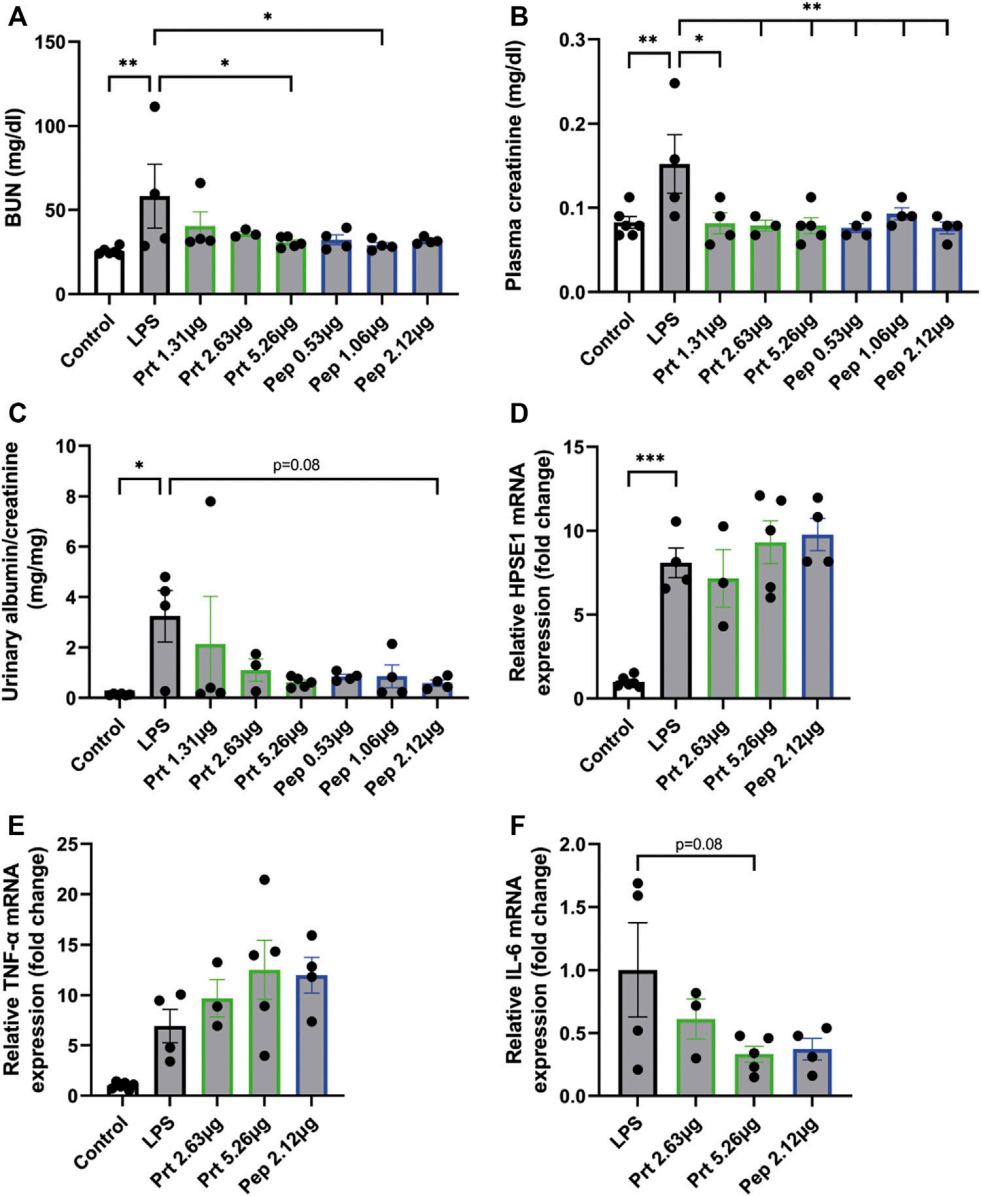
HPSE2 protein and peptides treatment have beneficial effects in LPS-induced glomerulonephritis **(A)**. BUN, **(B)**. plasma creatinine, and **(C)**. Urinary albumin/creatinine ratio of mice injected with LPS (2 days) and treated with different concentrations of HPSE2 protein or HPSE2 peptide mix **(D)**. Cortical HPSE1, **(E)**. cortical TNF-α, **(F)**. cortical IL-6 mRNA expression of mice injected with LPS (2 days) and treated with different concentrations of HPSE2 protein or HPSE2 peptide mix of 381–410, 391–420, and 401–430. Data are expressed as mean ± SEM. *n* ≥ 4 *p < 0.05, **p < 0.01, ***p < 0.001. HPSE2, heparanase-2; HPSE1, heparanase-1; Prt, His-mHPSE2 protein; pep, HPSE2 peptide mix of 381–410, 391–420, and 401–430; BUN, Blood urea nitrogen plasma level.

To further explore the therapeutic potential of HPSE2, the HPSE2 protein and a mix of the HPSE2 peptides 381–410, 391–420, and 401–430 were tested as inhibitors in the LPS-induced glomerulonephritis mouse model. Both HPSE2 protein and mix of HPSE2 peptides significantly ameliorated the effect of LPS on renal outcome as measured by blood urea nitrogen (BUN) (Figure 5A), plasma creatinine levels (Figure 5B), and urinary albumin creatinine ratio (Figure 5C). The HPSE2 protein even showed a dose response inhibitory effect at the BUN and urinary albumin creatinine ratio.

Notably, cortical HPSE1 and TNF-α mRNA expression were unaltered (Figures 5D, E), which indicates that HPSE2 protein and peptides do not inhibit the LPS-induced inflammatory response, but only the subsequent effect on kidney function. On the contrary, IL-6 showed a decrease in mRNA expression levels (Figure 5F). These data suggest that both HPSE2 protein and peptides have a protective effect on LPS-induced inflammatory conditions and subsequent glomerulonephritis *in vivo*.

### HPSE2 protein ameliorates the induction of diabetic nephropathy *in vivo*

**FIGURE 6:**
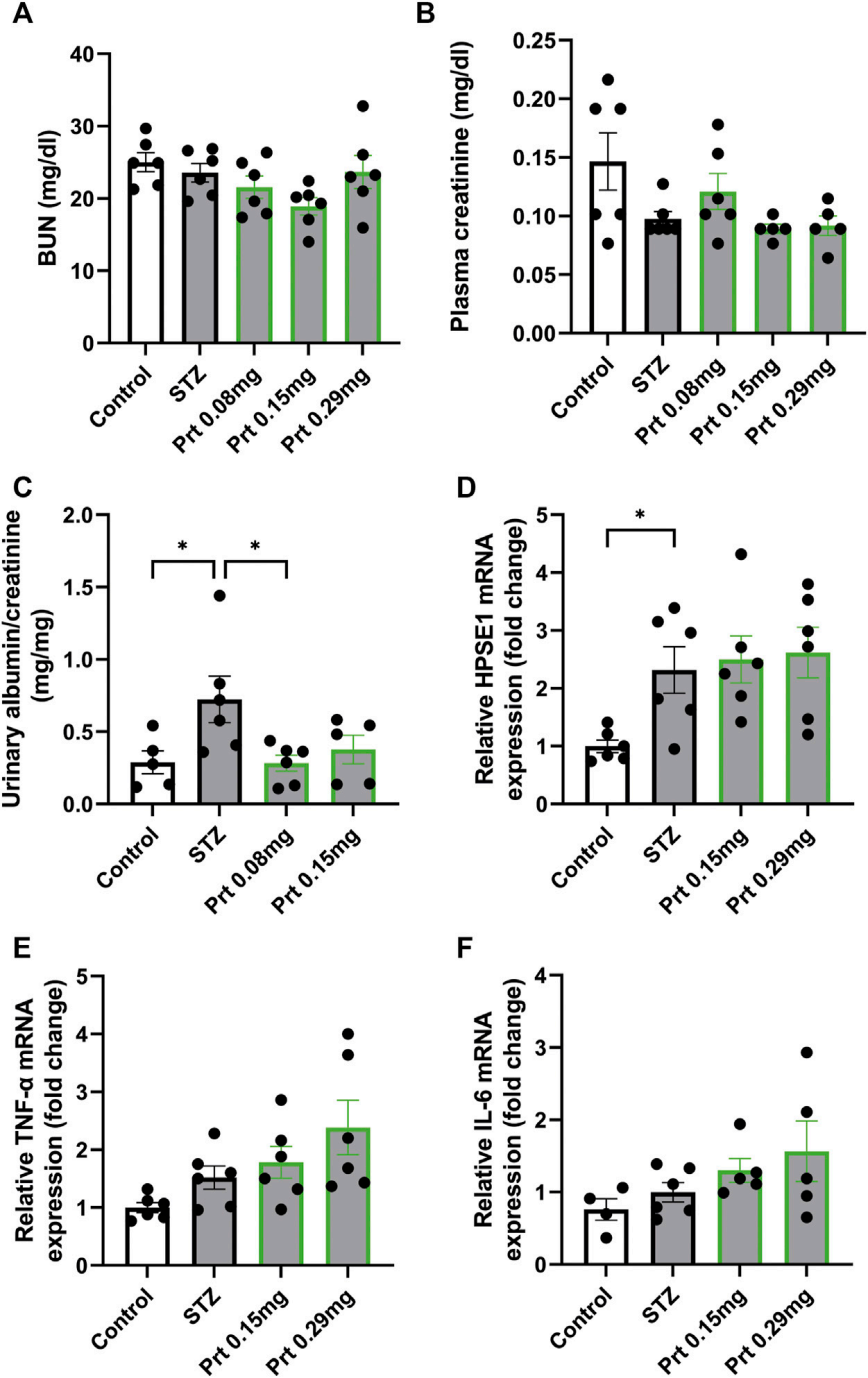
HPSE2 protein and peptides treatment beneficially affect experimental STZ-induced diabetic nephropathy **(A)**. BUN, **(B)**. plasma creatinine, and **(C)**. Urinary albumin/creatinine ratio of mice injected with STZ (12 weeks) and treated with different concentrations of HPSE2 protein or HPSE2 peptide mix **(D)**. Cortical HPSE1, **(E)**. cortical TNF-α, **(F)**. cortical IL-6 mRNA expression of mice injected with STZ (12 weeks) and treated with different concentrations of HPSE2 protein or HPSE2 peptide mix. Data are expressed as mean ± SEM. *n* ≥ 5 *p < 0.05. HPSE2, heparanase-2; HPSE1, heparanase-1; Prt, His-mHPSE2 protein; pep, peptide mix; BUN, Blood urea nitrogen plasma level; STZ, streptozotocin.

Similarly, we tested the potential protective effect of HPSE2 protein in the context of experimental DN. We performed this experiment with full-length HPSE2 protein only and not with the HPSE2 peptides because of the long experimental disease model and continuous administration of HPSE2. Whereas no clear differences in BUN and plasma creatinine values could be observed between the control mice and STZ-induced diabetic mice (Figures 6A, B), urinary albumin creatinine ratio was increased in STZ-induced diabetic mice compared to wild-type mice and significantly ameliorated by addition of HPSE2 protein (Figures 6B, C).

Moreover, cortical HPSE1 and TNF-α mRNA expression were unaltered (Figures 6D, E) like in the LPS-induced glomerulonephritis model. Unlike our findings in glomerulonephritis, IL-6 mRNA did not show a significant difference between STZ-induced diabetic mice with or without HPSE2 protein treatment (Figure 6F). Nevertheless, these results confirm our findings in LPS-induced glomerulonephritis and suggest that the protective effect of HPSE2 might be applicable for other glomerular diseases in which HPSE1 activity has been shown to play a role.

## Discussion

In this study, we explored the regulation of HPSE2 expression and the link between HPSE1 and HPSE2 regulation under inflammatory and diabetic conditions, to the best of our knowledge, for the first time. Furthermore, we showed the potential of full-length HPSE2 protein and HPSE2-derived peptides to inhibit HPSE1 activity, and we demonstrated a protective effect of HPSE2 protein and peptides in experimental glomerulonephritis and DN.

Our results on HPSE2 expression regulation contribute to the current body of knowledge about HPSE2 expression regulation. Getting a better understanding of the regulation of the natural HPSE1 inhibition by HPSE2 is valuable, since HPSE1 plays a prominent role in the manifestation of various glomerular diseases (van der Vlag and Buijsers, 2020; Rabelink et al., 2017; van den Hoven et al., 2007). In our study, HPSE2 expression was downregulated in anti-GBM and LPS-induced glomerulonephritis, streptozotocin-induced DN, and adriamycin nephropathy, whereas this effect on HPSE2 expression was absent upon treatment with the HPSE1 inhibitor SST001 and in HPSE1-deficient mice. These results are in line with previously reported data by Kiyan et al. in which decreased expression of HPSE2 was detected in serum and kidney medullary capillaries of mouse CLP polymicrobial sepsis model. A negative feedback mechanism of HPSE1 on HPSE2 expression under inflammatory conditions can be suggested based on our data on HPSE2 expression in combination with the knowledge that HPSE1 upregulation is associated with inflammatory diseases.

Remarkably, both HPSE2 protein and peptides improved kidney function in LPS-induced glomerulonephritis, while HPSE1 and TNF-α mRNA expression levels remained unaltered. Glomerulonephritis is characterized by the influx of inflammatory cells, proteinuria, and an impaired renal function. In previous studies, we have shown that although decay in renal function develops 2 days upon LPS injection, the influx of immune cells arises at an earlier stage in the development of glomerulonephritis (Garsen et al., 2016a). This phenomenon could explain the preserved increase in HPSE1 and TNF-α mRNA expression. Due to the experimental design of the study, mice got the HPSE2 protein and peptides 6 hours after induction of the experimental model, which might have been sufficient time to initiate the immune response and induce HPSE1 and TNF-α mRNA expression. In contrast to TNF-α, IL-6 mRNA expression levels were near-significantly decreased by addition of HPSE2 in experimental glomerulonephritis but not in DN. However, we were unable to detect IL-6 mRNA expression levels in control mice for the LPS study, which complicates the interpretation of the magnitude and thus the impact of the observed decreased IL-6 expression.

Since HPSE1 is also involved in inflammation (Goodall et al., 2014; Lever et al., 2014), the increased HPSE1 and TNF-α expression might be prevented if HPSE2 is administered concomitant with LPS at induction of experimental glomerulonephritis. This effect has been shown previously by Kiyan et al. in the CLP sepsis mouse model. They reported decreased plasma levels of IL-6 and TNF-α in mice induced with LPS co-injected with full-length HPSE2 protein compared to LPS alone already upon 2 h. Unfortunately, no assessment of cytokine or HPSE1 mRNA expression in the renal cortex or other tissues was reported, which makes it difficult to directly compare our observations with their findings.

The HPSE2 peptides that showed most potential in the first screening experiment can deepen our understanding about the mechanisms behind HPSE1 inhibition mediated by HPSE2. We showed that besides the full-length HPSE2 protein also specific HPSE2-derived peptides can be effective in inhibition of HPSE1 activity and the treatment of glomerular diseases. For instance, knowledge about previously annotated functions of a specific domain of a protein can provide such information. Although no functional domains or regions are annotated for HPSE2, the structural similarity between HPSE2 and HPSE1 might provide more insight in HPSE1 inhibitory characteristic of HPSE2. The two most effective peptides in HPSE1 inhibition, HPSE2 peptide 381–410 and 401–430, both possess a sequence of three amino-acids that are annotated as HS-binding domain in HPSE1. Notably, another research group has suggested that HPSE2 inhibits HPSE1 activity by interfering with the interaction between HPSE1 and HS due to the binding affinity of HPSE2 with HS (Levy-Adam et al., 2010).

Other HPSE1 inhibitors have been tested both *in vitro* and *in vivo* for their application in glomerular diseases as well. Administration of HPSE1 substrates, such as soluble HS, heparin, and low-molecular-weight heparins, all comprise the potential to reduce albuminuria. One example of such class of drugs is sulodexide, which is a highly purified mixture that consists for 80% of low-molecular-weight heparin and for 20% of dermatan sulfate (Poplawska et al., 1997). Sulodexide was shown to be effective in restoring the glycocalyx thickness and showed a trend towards normalization of systemic albumin clearance in a study of type 2 diabetes mellitus patients. However, a contradicting effect was observed in type 2 diabetes mellitus and diabetic nephropathy patients in two other studies (Rabelink et al., 2017). Furthermore, PI-88 has been reported as an effective HPSE1 inhibitor in animal models for glomerular diseases, such as DN (Simeonovic et al., 2013). Inhibition of HPSE1 activity by the specific HPSE1 inhibitor SST0001 in type 1 diabetic wild type mice also resulted in less albuminuria and better renal function compared to vehicle treated diabetic mice (Gil et al., 2012; Garsen et al., 2014).

Thus far, only one study has shown the implication of HPSE2 as a preventive strategy in inflammation, but this study reported two markers only, and no effect on outcome of disease manifestation was demonstrated. Since we were able to show a positive effect of HPSE2 treatment on renal function in experimental models for glomerular diseases, our data strongly adds to the current body of literature on the promise of HPSE2 as a therapeutic in glomerular diseases.

There are also some limitations in our study. The effect of HPSE2 treatment on kidney function was evident even though the number of mice per group was relatively low. Since only half of the mice got ill upon LPS injection, this is a limitation. Furthermore, STZ-induced diabetic mice showed increased albumin-creatinine ratio, whereas no clear differences in BUN and plasma creatinine values could be observed between the control mice and STZ-induced diabetic mice, suggesting the model was relatively mild. In addition, we did not perform a toxicology or pharmacological distribution study, which would give more insight in where the HPSE2 protein and peptides are distributed to *in vivo* and therefore associated with the mode of action of HPSE2. Although we were already able to show a beneficial effect of HPSE2, the toxicology assessment and pharmacological distribution should be addressed in future studies.

In conclusion, we found that HPSE2 expression was downregulated in experimental glomerular disease models for glomerulonephritis, diabetic nephropathy and adriamycin nephropathy, a model for FSGS, which is suggested to be linked to the increased HPSE1 activity in these diseases. Furthermore, both HPSE2 protein and HPSE2 peptides showed great promise in preventing loss of renal function in experimental glomerulonephritis and DN. Although our initial results indicate that HPSE2 inhibits HPSE1 activity and prevents decline of renal function by competitively binding to HS, modelling of HPSE2 and mutation studies could further detail the mechanism of HPSE1 activity inhibition by HPSE2, which might ultimately contribute to better treatment options for patients who suffer from glomerular diseases.

## Data Availability

The datasets presented in this study can be found in online repositories. The names of the repository/repositories and accession number(s) can be found in the article/Supplementary Material.

## Supplementary material

SUPPLEMENTARY FIGURE S1 Production of HPSE2 protein with in vitro translation **(A)**. Final construct for production of HPSE2 protein **(B)**. Western blot detection of HPSE2 protein with anti-HPSE2 antibody after the His tag mediated affinity purification **(C)**. Western blot detection of HPSE2 protein with anti-HIS antibody.

SUPPLEMENTARY FIGURE S2 HPSE2 is downregulated in experimental adriamycin nephropathy **(A)**. Renal cortical HPSE2 mRNA expression of wild-type mice treated with or without adriamycin for 10 or 23 days. *n* ≥ 5 **(B)**. Renal cortical HPSE2 protein expression of wild-type mice treated with or without adriamycin for 10 or 23 days. *n* ≥ 5 *p < 0.05, **p < 0.01. HPSE2, heparanase-2; AU, arbitrary units; Adria, adriamycin.
