# Supplementary figures and images for: Heparanase-2 protein and peptides have a protective effect on experimental glomerulonephritis and diabetic nephropathy
Source: Front Pharmacol. 2023 Apr 27;14:1098184. doi: 10.3389/fphar.2023.1098184 (PMC10172501; doi:10.3389/fphar.2023.1098184)

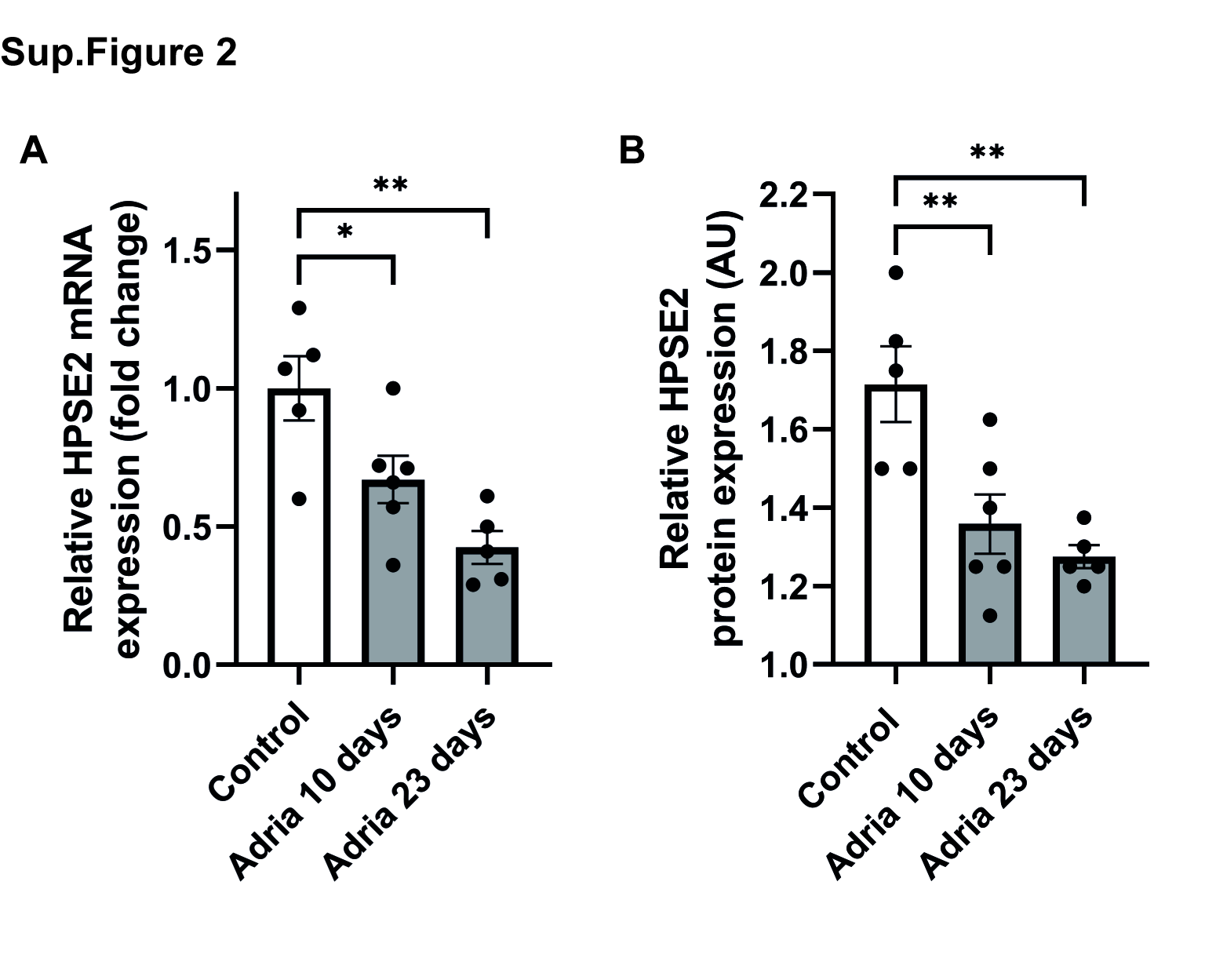

Supplement: Supplementary file 1 [file Image2.TIF]

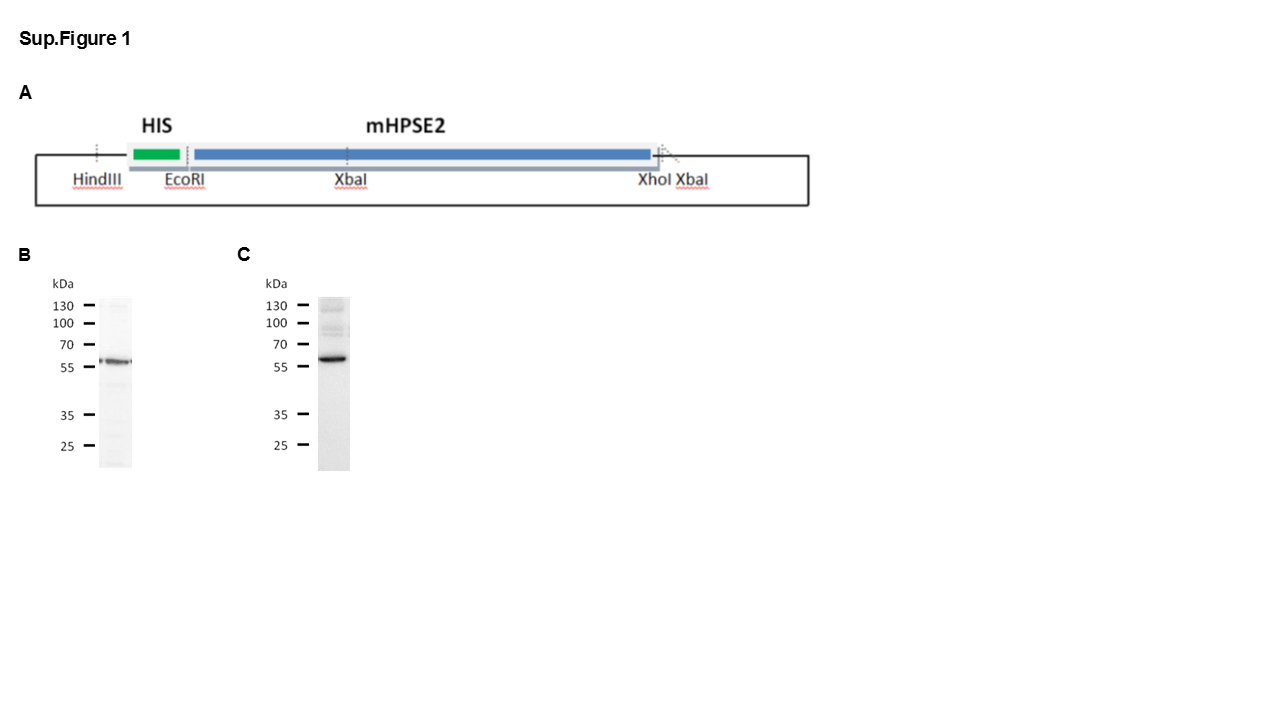

Supplement: Supplementary file 2 [file Image1.TIF]
